# Antiproliferative and Antioxidant Activities of Two Extracts of the Plant Species *Euphorbia dendroides* L.

**DOI:** 10.3390/medicines5020036

**Published:** 2018-04-20

**Authors:** Agena Ghout, Amar Zellagui, Noureddine Gherraf, Ibrahim Demirtas, Yaglioglu Ayse Sahin, Meriem Boukhenaf, Mesbah Lahouel, Gema Nieto, Salah Akkal

**Affiliations:** 1 Laboratory of Biomolecules and Plant Breeding, Life Science and Nature Department, Faculty of Exact Science and Life Science and Nature, University of Larbi Ben Mhidi, Oum El Bouaghi 04000, Algeria; agenaghout@yahoo.fr (A.G.); zellaguia@yahoo.com (A.Z.); 2 Department of Chemistry, Faculty of Exact Science and Life Science and Nature, University of Larbi Ben, Mhidi Oum El Bouaghi 04000, Algeria; ngherraf@yahoo.com; 3 Laboratory of Plant Research, Department of Chemistry, Faculty of Science, Uluyazi Campus, Cankiri Karatekin University, Cankiri 18100, Turkey; ibdemirtas@gmail.com (I.D.); aysesahin1@gmail.com (Y.A.S.); 4 Department of Pathological Anatomy, University Hospital Center of Constantine, Constantine 25000, Algeria; Boukhenaf.Meriem@gmail.com; 5 Laboratory of Molecular Toxicology, University of Jijel, Jijel 18000, Algeria; lahouelmesbah@yahoo.fr; 6 Department of Food Technology, Nutrition and Food Science, Faculty of Veterinary Sciences, University of Murcia, Campus de Espinardo, Espinardo, Murcia 30100, Spain; gnieto@um.es; 7 Laboratoire de Phytochimie et Analyses Physico-Chimiques et Biologiques, Université Mentouri de Constantine, Route de Aïn El Bey, Constantine 25000, Algeria

**Keywords:** *Euphorbia dendroides*, phenolic compounds, HPLC, antioxidant activity, antiproliferative activity

## Abstract

**Background:** These days, the desire for naturally occurring antioxidants has significantly increased, especially for use in foodstuffs, cosmetics, and pharmaceutical products, to replace synthetic antioxidants that are regularly constrained due to their carcinogenicity. **Methods**: The study in hand aimed to appraise the antioxidant effect of two *Euphorbia dendroides* extracts using reducing power, anti-peroxidation, and DPPH (1,1 Diphenyl 2 Pycril Hydrazil) scavenging essays, in addition to the anticancer activity against two tumor cell lines, namely C6 (rat brain tumor)cells, and Hela (human uterus carcinoma)cell lines. **Results**: The results indicated that the ethyl acetate extract exhibited antiradical activity of 29.49%, higher than that of *n*-butanol extract (18.06%) at 100 µg/mL but much lower than that of gallic acid (78.21%).The ethyl acetate extract exhibits better reducing capacity and lipid peroxidation inhibitory activity compared to *n*-butanol extract but less than all tested standards. Moreover, the ethyl acetate extract was found to have an antiproliferative activity of more than 5-FU (5-fluoro-Uracil) against C6 cells at 250 µg/mL with *IC*_50_ and *IC*_75_ of 113.97, 119.49 µg/mL, respectively, and good cytotoxic activity against the Hela cell lines at the same concentration. The HPLC-TOF-MS (high performance liquid chromatography-Time-of-flight-Mass Spectrometry) analyses exposed the presence of various compounds, among which Gallic and Chlorogenic acids functioned as major compounds. **Conclusions**: The two extracts exhibited moderate anticancer abilities and behaved somewhat as average antioxidant agents. Based on the total phenolics and flavonoids contents, as well as HPLC results, it could be concluded that antiproliferative and antioxidant activities depend upon the content of different phenolics and flavonoids.

## 1. Introduction

The free radicals are chemical compounds, which involve in their structure one or more impaired electrons, making them extremely unstable and hence predisposing them to extract electrons from other compounds to reach stability. Oxidative stress refers to an imbalance between the production of the free radicals and the antioxidant defense system. Oxidative stress is regarded as substantial in the initiation and expansion of many recent ailments including irritation, cataracts, tumors, and autoimmune and neurodegenerative disorders [1,2,3].

At present, the interest in naturally antioxidants has noticeably increased for use in foodstuffs and cosmetic and pharmaceutical ingredients as a substitute for artificial antioxidants that are steadily limited due to their suspicious carcinogenic effect [4,5]. Plants are viewed as a prospective source of natural antioxidants, especially the secondary metabolites such as phenolics, flavonoids, and terpenes, which are formed by plants to support growth under unfavorable conditions [6]. These herbs represent crucial ingredients of folk medicine applied throughout the world owing to their low price, effortless access, and inherited practice [7]. Lately, phenolics have received extensive awareness because of their physiological role, including as antioxidants and in antimutagenic and antitumor activities [8]. *Euphorbia,* one of the most various genus of flowering plants and the largest genus of the *Euphorbiaceae*, comprises about 2000 species [9,10]. The frequent name “spurge” derives from the French word espurgier (latinexpurgare), which denotes “to purge” due to the use of Euphorbia latex as purgative. The Botanical name Euphorbia derives from the Greek *Euphorbus* [11]. The compounds extracted from this genus comprise flavonoids, triterpenoids, amino acids, and alkaloids [12]. *Euphorbia* is reported to possess inflammatory, antiarthritic, antiamoebic, spasmolytic, antiviral, hapatoprotective, and antitumor activity [13,14,15,16]. Some species are used in folk medicine to treat skin diseases, gonorrhea, migraines, intestinal vermins, and warts [17,18].

*Euphorbia dendroides* encloses rich ethnopharmacological properties already documented in the old medical literature; it was used to remove warts and as a fish poison [19]. It was reported as well to contain many bioactive chemical compounds such as Jatrophane Esters [20]. 

The study in hand aims to assess the in vitro antioxidant and antiproliferative effects of the ethyl acetate and *n*-butanol extracts of the aerial parts of *Euphorbia dendroides* grown in Algeria.

## 2. Materials and Methods

### 2.1. Chemicals

Folin-ciocalteu reagent, anhydrous sodium carbonate (Na_2_CO_3_), Gallic acid, Ascorbic acid, Aluminum chloride (AlCl_3_), 2,2-diphenyl-1-picrylhydrazyl radical (DPPH), quercetin, potassium ferric cyanide (K_3_Fe(CN)_6_), iron (III) chloride (FeCl_3_), methanol, ethyl acetate, *n*-hexane, *n*-butanol, ethanol, trichloroacetic acid(TCA), thiobarbituric acid (TBA), HCl, NaOH, FeSO_4_, Tween 20, phosphate buffer, and BrdU Cell Proliferation ELISA assay reagent were provided by Roche, Berlin, Germany. Dulbecco’s modified eagle’s medium was purchased from DMEM, Sigma, Munich, Germany. Fetal bovine serum, PenStrep solution, and 5-fluorouracil were purchased from Sigma, Munich, Germany.

### 2.2. Plant Material

The above ground part of the plant was collected from Bejaia (Cap-Carbon) during May 2013 (flowering stage). Dr. Rebbas from the Department of botany, M’sila, Algeria identified the plant. A voucher sample was deposited in the Laboratory of Biomolecules and Plant Breeding, University of Larbi Ben M’hidi Oum El Bouaghi, Algeria under the voucher number ZA 140.

### 2.3. Extraction 

The extraction process was carried out according to the method of Wang [21]. The plant was air-dried and preserved at ambient temperature and then crushed into powder. 200 g of plant was soaked in 500 mL of 70% methanol-water for 1 week at room temperature and filtered using Whatman paper N°1. The extraction process was repeated three times and the hydroalcoholic solutions were collected, filtered, and dried under vacuum. The residue was suspended in 200 mL of distilled water and partitioned in ethyl acetate (3 × 150 mL) and *n*-butanol (3 × 150 mL). The resulting solutions were concentrated in vacuum, to yield the following fractions: EtOAc (0.84 g), *n*-butanol (4.5 g). The fractions were kept at 4 °C in the dark until further analysis.

### 2.4. HPLC-TOF/MS Analysis

Phenolic content of the plant extract was determined using Agilent Technology of 1260 Infinity HPLC System (Agilent Technologies, Santa Clara, CA, USA)joined with 6210 Time of Flight (TOF) LC/MS detector (Agilent Technologies, Santa Clara, CA, USA) and ZORBAX SB-C18 (4.6 × 100 mm, 3.5 μm) column (Agilent Technologies, Santa Clara, CA, USA). Mobile phases A and B were ultra-pure water with 0.1% formic acid and acetonitrile, respectively. Flow rate was 0.6 mL min^−1^, and column temperature was 35 °C. Injection volume was 10 μL. The solvent rate was 0–1 min 10% B, 1–20 min 50% B, 20–23 min 80% B, 23–25 min 10% B, and 25–30 min 10% B. Determination of the phenolic compounds was performed by comparison of the standard compounds with the samples in terms of retention times and *m*/*z* values. Ionization mode of HPLC-TOF/MS instrument was negative and operated with a nitrogen gas temperature of 325 °C, nitrogen gas flow of 10.0 L min^−1^, nebulizer of 40 psi, capillary voltage of 4000 V, and, finally, fragmentor voltage of 175 V. For sample analysis, dried crude extracts (200 ppm) were dissolved in methanol at room temperature. Samples were filtered passing through a PTFE (0.45 μm) filter by an injector to remove particulates.

### 2.5. Phenolic Contents Analysis

#### 2.5.1. Total Phenolic Content (TPC)

The Total phenolic content (TPC) of both extracts was determined using the Folin-Ciocalteau Reagent (FCR) according to the method of Singleton [22] and external calibration with Gallic acid. Briefly, 0.5 mL of the diluted solution of each extract in methanol was added to 2.5 mL of FCR (diluted 1/10 with distilled water) and mixed. After 5 min, 2 mL of sodium carbonate aqueous solution Na_2_CO_3_ (75 g/L) was added to the mixture and incubated at 40 °C for 30 min. The absorbance was measured at 760 nm. Results are presented as mg of Gallic acid equivalent (GAE)/g of dry extract using Gallic acid calibration curve. All experiments were performed in triplicate, and the results were averaged.

#### 2.5.2. Total Flavonoids Content (TFC)

Flavonoids as among the most varied and prevalent group of natural compounds are probably the most important natural phenolics [23]. In this study, total flavonoids content of the two extracts was assessed following the aluminum Chloride colorimetric method [5].This method stands on the formation of a flavonoid-aluminum complex having λ_max_ at 430 nm. 1 mL of methanol extract was mixed with 1 mL of 2% AlCl_3_ methanol solution, and then the absorbance was determined at 430 nm using UV-VIS spectrophotometer. Total flavonoids content was expressed as mg quercetin equivalent (QE) per g of dry extract. All tests were done in triplicates.

### 2.6. Determination of Antioxidant Activity

#### 2.6.1. DPPH Radical Scavenging Assay

The radical scavenging activity of the two extracts was measured against DPPH (2,2-Diphenyl-1-picryl hydrasyl radical was measured according to the method of Masuda [24]). DPPH is a stable free radical, which on reaction with an antioxidant reduces from a violet color to the yellow-colored diphenyl-picryl-hydrazine. The free radical DPPH absorbs at 517 nm, but after reduction by an antioxidant the absorption decreases. Briefly, 20 µL of each extract (different concentrations, *w*/*v*) was mixed with 2 mL of methanol solution of DPPH radical (10^−4^ M). The mixture was shaken vigorously, and the absorbance values were read at 517 nm after incubation for 20 min in dark at room temperature. 

The radical scavenging activity was calculated using the following formula:

% inhibition = {[A_b_ − A_a_]/A_b_} × 100

Quercetin, gallic acid, and ascorbic acid were used as the positive control. The experiment was performed in triplicate.

#### 2.6.2. Reducing Power Assay

In this test, the yellow color of the test solution shifts to green reflecting the reducing power of the sample. The reducing agents in the solution initiate the reduction of the Fe^3+^/ferricyanide complex to the ferrous structure. Therefore, Fe^2+^ can be observed by measurement of the absorbance at 700 nm [25].

The reducing power of both extracts was determined according to the method of Oyaizu [26]. Briefly, 1mL of serial concentrations of the extract (20–100 µg/mL) was mixed with 1 mL of sodium phosphate buffer (0.2 mol dm^−3^, pH = 6.6) and 1 mL of potassium ferricyanide (1%). Reaction mixture was incubated at 50 °C for 20 min, and then 1 mL of trichloro-acetic acid (10%) was added and centrifuged for 10 min. From the upper layer, 1 mL was mixed with 1 mL of distilled water and 0.3 mL of FeCl_3_ (0.1%). Absorbance of resulting solution was measured at 700 nm. Quercetin, gallic acid, and ascorbic acid were used as standards. Values are presented as mg quercetin equivalent per g of extract.

#### 2.6.3. The Inhibition of Linoleic Acid Peroxidation

The antiperoxidation test of both extracts was realized using thiobarbituric acid (TBA) method, which is based on inhibition of linoleic acid peroxidation in accordance with the method of Choi [27].

This method was adopted for the lipid peroxidation test, with linoleic acid as the source of linoleic acid in an oxidation system catalysed by Fe-ascorbate. The samples (50–500 μg/mL) were mixed with linoleic acid solution (0.28 mg of linoleic acid and 0.28 mg of tween-20) in 100 μM phosphate buffer (500 mL of phosphate buffer (100 μM, pH = 7.4) and 150 μL of ascorbic acid (10 μM). The mixture was stirred and sonicated to give a homogeneous suspension solution. The linoleic acid peroxidation test started with the addition of 0.1 mL FeSO_4_ (10 μM) and was incubated at 37 °C for 60 min. The reaction mixture was cooled and added to 1.5 mL of trichloroacetic acid (10% in 0.5% HCl). Then, 3 mL TBA (1%, in 50 mM NaOH) was added. The reaction mixture and TBA solution were heated in the water bath at 90 °C for 60 min. After cooling down, 2 mL aliquots were taken from each sample and mixed with 2 mL of *n*-butanol and centrifuged at 1000×*g* for 30 min. The upper layer solution was separated for the spectroscopic measurement, and the absorbance of thiobarbituric acid-reacting substances (TBARS) in the supernatant were read at 532 nm and converted into the percentage of antioxidant activity. 

The percentage of linoleic acid peroxidation inhibition was defined as (%) = [(A_o_ − A_1_)/A_o_] × 100, in which A_o_ is the absorbance of control reaction (containing all reagents except the sample) and A_1_ is the absorbance of the sample or the standard.

### 2.7. Determination of In Vitro Antiproliferative Activity

Antiproliferative activity was evaluated by estimation of the inhibitory effect of the phenolics on the growth of cells on C6 (rat brain tumor) using proliferation BrdU ELISA assay, and was tested for HeLa cell lines using a real-time cell analyzer (xCELLigence) [28,29].

#### 2.7.1. Cell Culture

The cells were developed in Dulbecco’s modified eagle’s medium (DMEM, Sigma, Munich, Germany), complemented with 10% (*v*/*v*) fetal bovine serum (Sigma, Munich, Germany) and PenStrep solution (Sigma, Munich, Germany) at 37 °C in a 5% CO_2_ humidified atmosphere. 

#### 2.7.2. Cell Proliferation Assays

● *ELISA Assay*

The cells were laminated in 96-well culture plates (COSTAR, Corning, Oneonta, NY, USA) at a density of 30,000 cells per well. The samples activities were investigated at 250, 100, and 50 µg/mL. 5-FU was used as standard. Afterwards, the cells were incubated all night before applying the BrdU Cell Proliferation ELISA assay reagent (Roche, Berlin, Germany) in accordance with the manufacturer’s method. The quantity of cell proliferation was read at 450 nm using a microplate reader (Awareness Chromate, Ramsey, MN, USA). 

Results were given as percentage of the inhibition, in which the optical density measured from vehicle-treated cells was considered to be 100% of proliferation. The stock solution of the extracts was prepared in dimethyl-sulfoxide (DMSO) and diluted with DMEM. DMSO final concentration is below 0.1% in all tests. 5-FU was used as standard compounds. Percentage of inhibition of cell proliferation was calculated as follows: inhibition percentage = [1 − (A_treatments_/_Avehicle control_)] × 100.

The half-inhibitory concentration (*IC*_50_) is a measure of the effectiveness of a compound in inhibiting a biological function. In this paper, *IC*_50_ and *IC*_75_ values were determined using *ED*_50_ in addition to V1.0. 

● *Xcelligence Assay*

A real-time cell analyzer–single plate (RTCA-SP) instrument (Roche Applied Science, Basel, Switzerland) was used to analyze the ability of extracts to induce cell growth of HeLa cell line. A newly developed electronic cell sensor array, the xCELLigence RTCA, was used with a recently published literature method at concentrations of 250, 100, and 50 μg/mL. All the measurements were done in 10 min intervals and triplicated [30].

● *Statistical analysis*

The in vitro results of anticancer activity are means ± SD of six measurements. Differences between groups were tested with ANOVA. *p* values of <0.01 were considered as significant and analyzed by SPSS (version 11.5 for Windows 2000, SPSS Inc., Chicago, IL, USA).

The results of scavenging activity and total phenolic compounds were performed from the samples reading mean ±SD (standard deviation) using EXEL 2003. All analyses were carried out in triplicates.

## 3. Results and Discussion

### 3.1. Extraction Yield

The yield of the two crude extracts was calculated and presented in Table 1 and expressed as the ratio of the dry weight of plant extract to the dry weight of the plant material. The extraction yield varies depending on the nature of the used solvent. The higher yield is obtained with *n*-butanol extract (2.12%) followed by ethyl acetate extract (0.42%).

### 3.2. Total Phenolic Compounds and Total Flavonoids 

A number of modern studies are evidence for phenolics as the most dominant antioxidant compounds [31]. Therefore, the amount of total phenolic compounds and flavonoids in the two extracts were evaluated (Table 2).

The total phenolics of the plant extracts measured by the folin-ciocalteu method are presented as gallic acid equivalent in milligrams per gram of extract (mg GAE/g). Total phenolic content was found to range from 164.25 to 929.51 mg GAE/g.

The extraction of phenolics in plant material is affected by their chemical environment, the extraction process, particle size, period, and conditions of storage, as well as the presence of intrusive substances [32], in addition to solvent polarity, which has diverse effects on total phenolics and antioxidant activities [33,34].

The peak amount of total flavonoids was obtained in ethyl acetate extract (26.04 mg QE/g) followed by *n*-butanol extract (12.16 mg QE/g), signifying that the extracts are very complex and hold many other polyphenols, or that the polymerization degree of the polyphenols in the samples is important.

### 3.3. High Performance Liquid Chromatography Analysis

The phenolic composition of the two extracts was performed using HPLC analysis. Ten compounds were detected in ethyl acetate extract including Gallic and Chlorogenic acids as major components representing 614.12 and 642.01 mg/kg of plant, respectively. *N*-butanol extract, in turn, contains fourteen compounds, among which Gallic and Chlorogenic acids represent, as well, the major components with 481.94 and 4505.78 mg/kg of plant, respectively. The compounds were identified by comparing their chromatographic characteristics (retention time (t_R_), mass spectra) with reference standards (Table 3). 

The HPLC revealed the presence Rutin only in Ethyl acetate extract. On the contrary, Resveratrol, Hesperidin, Salicylic acid, 4-hydroxybenzoic acid, and Cinnamic acid were detected only in *n*-Butanol extract.

### 3.4. Antioxidant Activity

#### 3.4.1. DPPH Radical Assay

**Figure 1:**
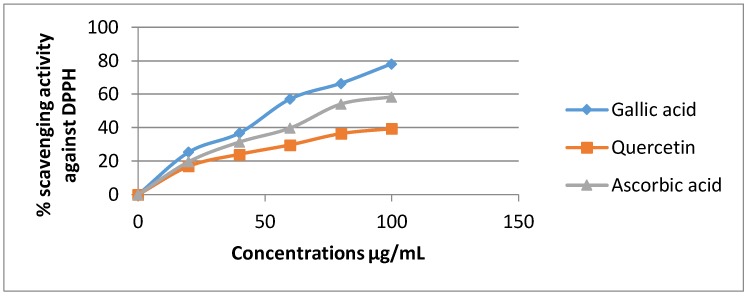
Inhibition of DPPH radical by the standards at different concentrations.

**Figure 2:**
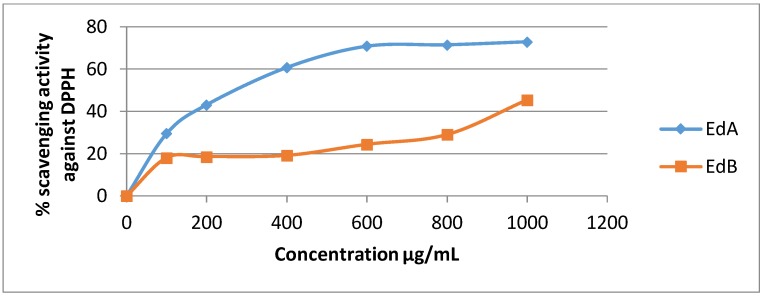
Inhibition of DPPH radical by the two extracts of *E. dendroides* at different concentrations.

The DPPH assay has been broadly used to estimate the free radical scavenging ability of natural compounds [35].In the presence of an antioxidant, DPPH radical obtains 1 more electron, and the absorbance decreases [36]. The degree of reduction in absorbance measurement is indicative of the radical scavenging strength of the extract. The two extracts of *Euphorbia dendroides* at different concentrations (100–1000 µg/mL) and the standards were tested for the scavenging effect on DPPH radical, and the inhibition percentage was balanced with those of quercetin, gallic acid, and ascorbic acid when used as standards. In this test, the two extracts of *E. dendroides* exhibited dose-dependent activities (Figure 1 and Figure 2). 

Gallic acid expressed the best antiradical activity (78.21%) compared to quercetin (39.45%) and ascorbic acid (58.41) at 100 µg/mL (Figure 1).

**Figure 3:**
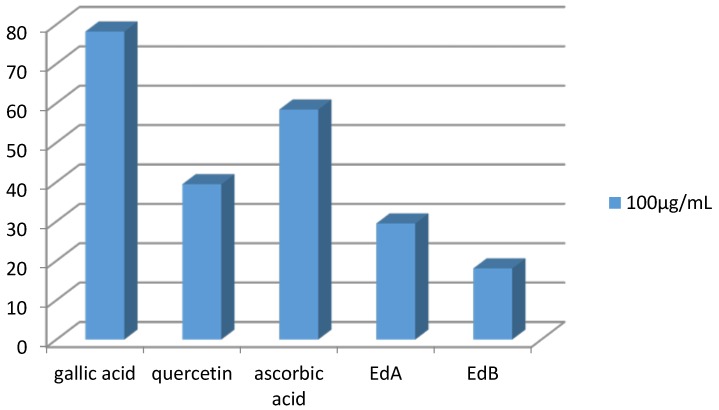
Comparison of DPPH inhibition between standards and both extracts at the same concentrations.

The Ethyl acetate extract demonstrated a moderate scavenger effect with inhibition rate of 29.49% at 100 µg/mL. In brief, the two extracts showed a lower antiradical activity than those exerted by positive controls (Figure 3).

Numeral reports on flavonoids, triterpenoids, and polyphenols designated that they acquire antioxidant and free radical scavenging activity [37]. Therefore, the presence of flavonoids and phenolics in the two extracts is probably responsible for the scavenging effects observed in this study. 

#### 3.4.2. Reducing Power Assay

Antioxidant activity was reported to be the development associated with reducing power [38]. Antioxidants decrease reactive radicals to stable species by producing electrons [21]. The reducing effect is a key parameter for estimation of antioxidant activity. The ferric reducing assay is simple, fast, and responsive for the antioxidant screening.

**Figure 4:**
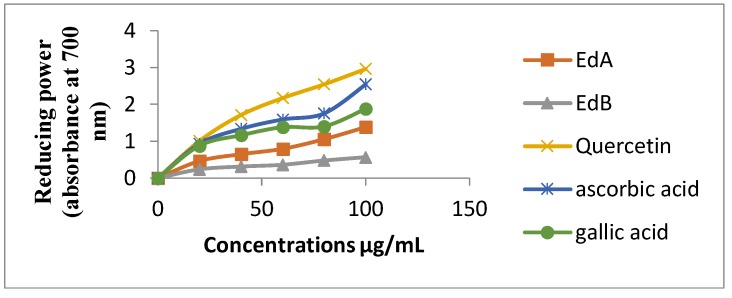
Reducing power of *E. dendroides* extracts and the standards at different concentrations.

The antioxidant activity of both extracts was evaluated by reducing power assay, based on the reduction of Fe^3+^/ferricyanide complex to the ferrous form in presence of antioxidants, over a concentration range of 20–100 µg/mL (Figure 4).A high absorbance value reveals that the sample has a stronger antioxidant activity [39].

The reducing power of the two extracts and standards on Fe^3+^ was found to be concentration-dependent. The two extracts exhibited varying reduction capacities. Ethyl acetate extract showed a higher reduction capacity with a value of 471.82 mg QE/g of extract. Both extracts had lower antioxidant activities than quercetin, gallic acid, and ascorbic acid when used as standards. The order of reducing capacity at 100 µg/mL is as follows: quercetin > ascorbic acid > gallic acid > ethyl acetate extract > *n*-butanol extract.

The antioxidant activities of many plant extracts increase with polyphenols content [39]. The reducing power activity follows the same order of the total flavonoids and total phenolics. The results of the antioxidant capacity of an extract depend greatly on the adopted methodology. Therefore, it is essential to compare diverse analytical methods varying in their oxidation initiators and targets in order to understand the biological activity of an oxidant [40,41,42,43].

#### 3.4.3. The Inhibition of Linoleic Acid Peroxidation

Lipid peroxidation is the oxidative degeneration of polyunsaturated lipids to make radical intermediates that cause oxidative damage. Lipid peroxidation (LP) acts as main agent in carcinogenesis [43]. Many cytolytic compounds such as reactive aldehyde products, including malondialdehyde (MDA), 4-hydroxy-2-nonenal (HNE), 4-hydroxy-2-hexenal (4-HHE), and acrolein are produced as metabolites of lipid peroxidation [44]. MDA has been frequently used as a suitable biomarker for lipid peroxidation. The thiobarbituric method establishes the amount of peroxide in the reaction medium, as peroxide is the major product generated in lipid peroxidation. This assay provides information about the aptitude of phenolics to make complex with Fe^++^ or antagonizes linoleic acid. The inhibition of linoleic acid is found to be dose dependent.

The standard BHT used in this assay exhibited the highest potency against non-enzymatic linoleic acid’s peroxidation with inhibition value of 86.65% at 500 µg/mL.

**Figure 5:**
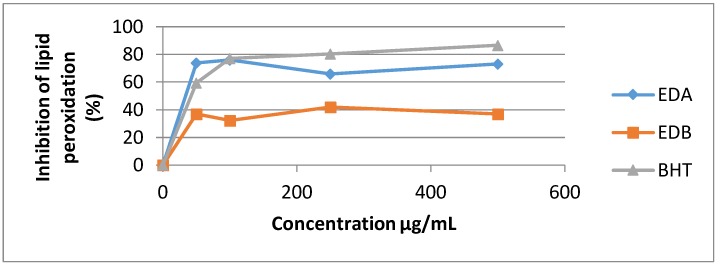
Percentage inhibition of linoleic acid peroxidation of the two *E. dendroides* extracts.

The Ethyl acetate extract illustrates good Lipid peroxidation inhibitory action (73.11% at 500 µg/mL) and *n*-butanol extract has the least potency (37.12% at 500 µg/mL) (Figure 5). 

### 3.5. Antiproliferative Activity

The discovery of new anticancer substances from nature is a current research tendency because of the minor toxicity of natural compounds [45]. Herbal medicine is being increasingly employed in the supervision of many cancer cures [46].

**Figure 6:**
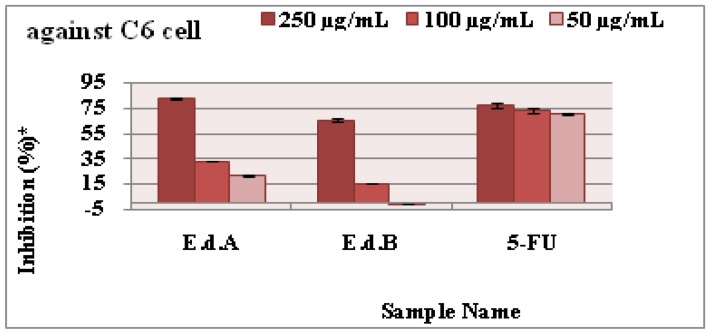
The antiproliferative activities of the two extracts against C6 cells. Each substance was tested twice in triplicates against cell lines. Data show average of two individual experiments (*p* < 0.01).

**Figure 7:**
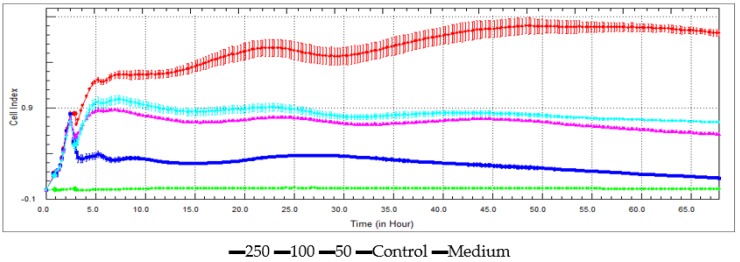
Antiproliferative activity against the Hela cell lines of ethyl acetate extract.

**Figure 8:**
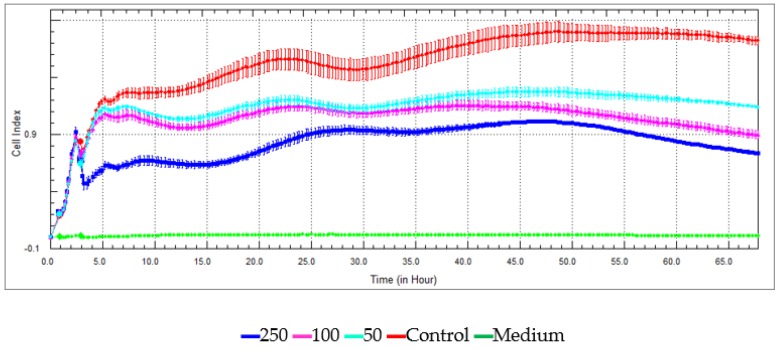
Antiproliferative activity against the Hela cell lines of *n*-butanol extract.

Antiproliferative assays of the two extracts and 5-FU were investigated on C6 cell lines using proliferation BrdU ELISA test and against HeLa cell lines using xCELLigence RTCA instrument at 250 µg/mL, 100 µg/mL, and 50 µg/mL. The antiproliferative effect increased with increasing dose against C6 cells (Figure 6) and Hela cells (Figure 7 and Figure 8). The IC_50_ and IC_75_ values are given in Table 4. The Ethyl acetate extract exhibits higher antiproliferative activities than 5-FU against C6 cells at 250 µg/mL (Figure 6). *N-butanol* extract, in turn, has a remarkable antiproliferative activity compared with the standard at 250 µg/mL.

Some studies on the antiproliferative effect of *E. helioscopia* on five human cancer cell lines (human hepatocellular carcinoma cell lines SMMC-7721, BEL-7402, HepG2, gastric carcinoma cell line SGC-7901, and colorectal cancer cell line SW480) were reported by Wang and revealed that *n*-butanol extract exerted no inhibitory effects on all cells. Nevertheless, ethyl acetate extract was very active at 200 µg/mL on SMMC-7721 cells for 72 h by exerting an inhibition rate of 80.91% on cell growth [36].

## 4. Conclusions

In light of the results obtained, it is concluded that the two extracts of *E. dendroides* display important antiradical, reducing capacity, and lipid peroxidation inhibition activities. Antiproliferative activity carried out against two cancer lines revealed that the ethyl acetate extract is more potent than the *n*-butanol extract. Based on the HPLC results, the two extracts were found to contain many important phenolic compounds that contribute to enhancing the studied activities. More studies are required to perform in vivo assays.

## Figures and Tables

**Table 1:** Extractive values of *Euphorbia dendroides* extracts.

Extract	Yield (%)
Ethyl acetate extract	0.42%
*n*-Butanol extract	2.12%

**Table 2:** Phenolic compounds of *Euphorbia dendroides* extracts.

Extract	TPC(mg GAE/g of Extract)	TF(mg QE/g of Extract)
Ethyl acetate	929.51 ± 20.1	26.04 ± 0.32
*n*-Butanol	164.25 ± 16.40	12.16 ± 0.2

TPC: total phenolic compounds, TF: total flavonoids.

**Table 3:** Composition of ethyl acetate and *n*-butanol extracts determined by HPLC-TOF/MS (mg of phenolic compound/kg plant).

Compounds	RT	EtOAc	*n*-Butanol
Gallic acid	2.831	614.12	481.94
Gentisic acid	4.358	33.61	1.25
4-hydroxybenzoic acid	5.531		9.35
chlorogenic acid	5.984	642.01	4505.78
protocatechuic acid	6.959	7.74	3.08
caffeic acid	7.623	67.79	2.51
Vanillic acid	7.796	10.88	4.05
Rutin	9.081	0.03	-
P-coumaric acid	9.917	11.64	10.07
chicoric acid	10.982	1.02	3.22
Ferulic acid	11.082	8.81	6.84
Hesperidin	12.284	-	1.49
Salicylic acid	13.284	--	9.70
Resveratrol	14.682	-	42.31
Cinnamic acid	15.779	--	10.70

**Table 4:** *IC*_50_ and *IC*_75_ values of the extracts against C6 cell.

Inhibition Concentration	Ethyl Acetate	*N*-Butanol
*IC*_50_ (µg/mL)	119.49	151.18
*IC*_75_ (µg/mL)	185.74	200.62
